# Transarterial embolization with n-butyl cyanoacrylate for the treatment of active abdominopelvic bleeding in the polytraumatized patient

**DOI:** 10.1186/s42155-021-00222-w

**Published:** 2021-05-06

**Authors:** Rafael Kiyuze de Freitas, Lucas Moretti Monsignore, Luis Henrique de Castro-Afonso, Guilherme Seizem Nakiri, Jorge Elias-Junior, Valdair Francisco Muglia, Sandro Scarpelini, Daniel Giansante Abud

**Affiliations:** 1 Division of Interventional Radiology, Department of Radiology, Hematology and Oncology, Medical School of Ribeirão Preto, University of São Paulo, Avenida Bandeirantes, 3900, Monte Alegre, Ribeirão Preto, SP 14048-090 Brazil; 2 Division of Abdominal Radiology, Department of Radiology, Hematology and Oncology, Medical School of Ribeirão Preto, University of São Paulo, Ribeirão Preto, Brazil; 3 Division of Emergency Surgery, Department of surgery and anatomy, Medical School of Ribeirão Preto, University of São Paulo, Ribeirão Preto, Brazil

**Keywords:** Hemorrhage, Trans-arterial embolization, Interventional radiology, Wounds and injuries

## Abstract

**Purpose:**

An increasing number of polytraumatized patient presenting with active abdominal pelvic bleeding (APB) have been treated by endovascular selective embolization. However, reports on evaluate the efficacy, safety and complications caused by this technique have been limited. The aim of this study was to assess the safety and efficacy of embolization of APB using N-butyl cyanoacrylate glue (NBCA).

**Materials and methods:**

Single center retrospective study, that included consecutive 47 patients presenting with traumatic APB treated by embolization with NBCA between January 2013 and June 2019. The efficacy endpoint was defined as the absence of contrast extravasation immediately after procedure and clinical stabilization in the following 24 h after procedure. Clinical stabilization was defined as no rebleeding after embolization or the need for a surgical approach until the patient is discharged. Safety endpoint were any technical or clinical complications related to the embolization procedure.

**Results:**

The mean age of patients was 38.6 years (3–81), with a predominance of males (87.2%). The major causal factor of APB being involvement in a car accident, accounting for 68% of cases. Of the 47 cases, 29.8% presented pelvic trauma and the remaining (70.2%) presented abdominal trauma. The efficacy rate was 100%, while no complications related to the procedure were observed. The mortality rate was 14.8% (7/47) due to neurologic decompensation and other clinical causes.

**Conclusion:**

Endovascular embolization of traumatic abdominopelvic bleedings appear to be a highly safe and effective treatment, while avoiding emergent exploratory open surgeries.

## Introduction

Injuries due to violence and accidents, called external causes, have a great social impact because of their high prevalence and incidence (Rezende Neta et al. 2012; Whitaker et al. 1998). Trauma is the 8th leading cause of death in the world’s population, and the first among the world’s young population (Global Health Observatory (GHO) data 2016), thus represents a major impact factor of global public health.

About 40% of early deaths related to polytrauma patients are caused by uncontrollable bleeding related to trauma, especially when hemorrhage originates from solid organs and pelvic fractures (Papakostidis et al. 2012; Lopera 2010; Ptohis et al. 2017; Hildebrand et al. 2006). Super selective transarterial embolization (SE) of injured vessels is an adjuvant method in the treatment of trauma patients and has broadened the spectrum of care to these patients. This technique can provide hemostasis in areas of difficult surgical access, providing non-surgical management of solid visceral lesions or isolated vascular lesions and quickly stop bleeding while preserve organ’s function (Ptohis et al. 2017; Wallis et al. 2010; Ierardi et al. 2016). Moreover, compared to the open surgery, embolization is associated to reduced physiological stress, reduced blood transfusions and volume resuscitation, and reduced mortality rates (Wallis et al. 2010; Ierardi et al. 2016; Coccolini et al. 2017).

The key embolic materials used in abdominalpelvic bleeding (APB) are N-butyl cyanoacrylate (NBCA), metal coils, hemostatic gelatin sponge (Gelfoam®), Amplatzer® plugs, or association between methods (Monsignore et al. 2012).

The main objective of this study was to assess the safety and efficacy of SE for traumatic APB using NBCA as a first line strategy.

## Materials and methods

A single-center retrospective study that included consecutive 47 patients with blunt or perforated abdominal-pelvic trauma with active bleeding as demonstrated by imaging methods and who underwent SE with NBCA, between January 2013 and June 2019. NBCA was selected as the embolic material due to its high availability, its low cost compared to other embolization materials, and the interventionists’ broad experience with this material. SE was indicated in conference between surgeons and interventional radiologists which indicated a first line embolization for patients presenting with APB and that had hemodynamic instability, or failure of conservative treatment, or active bleeding on imaging examination.

The assessed variables were gender, age, causal factor of the injury, site of the bleeding, type imaging finding, embolic material used. The efficacy endpoint was defined as two post-procedure parameters: (1) the absence of contrast extravasation immediately after procedure and (2) clinical stabilization in the following 24 h after procedure. Clinical stabilization was defined as no rebleeding after embolization or no need for a surgical approach until the patient discharged. Safety endpoint were absence of SE related technical or clinical complications. Technical complications were embolization other arteries not associated with the bleeding and/or any SE-related hemorrhaging.

**Fig. 1:**
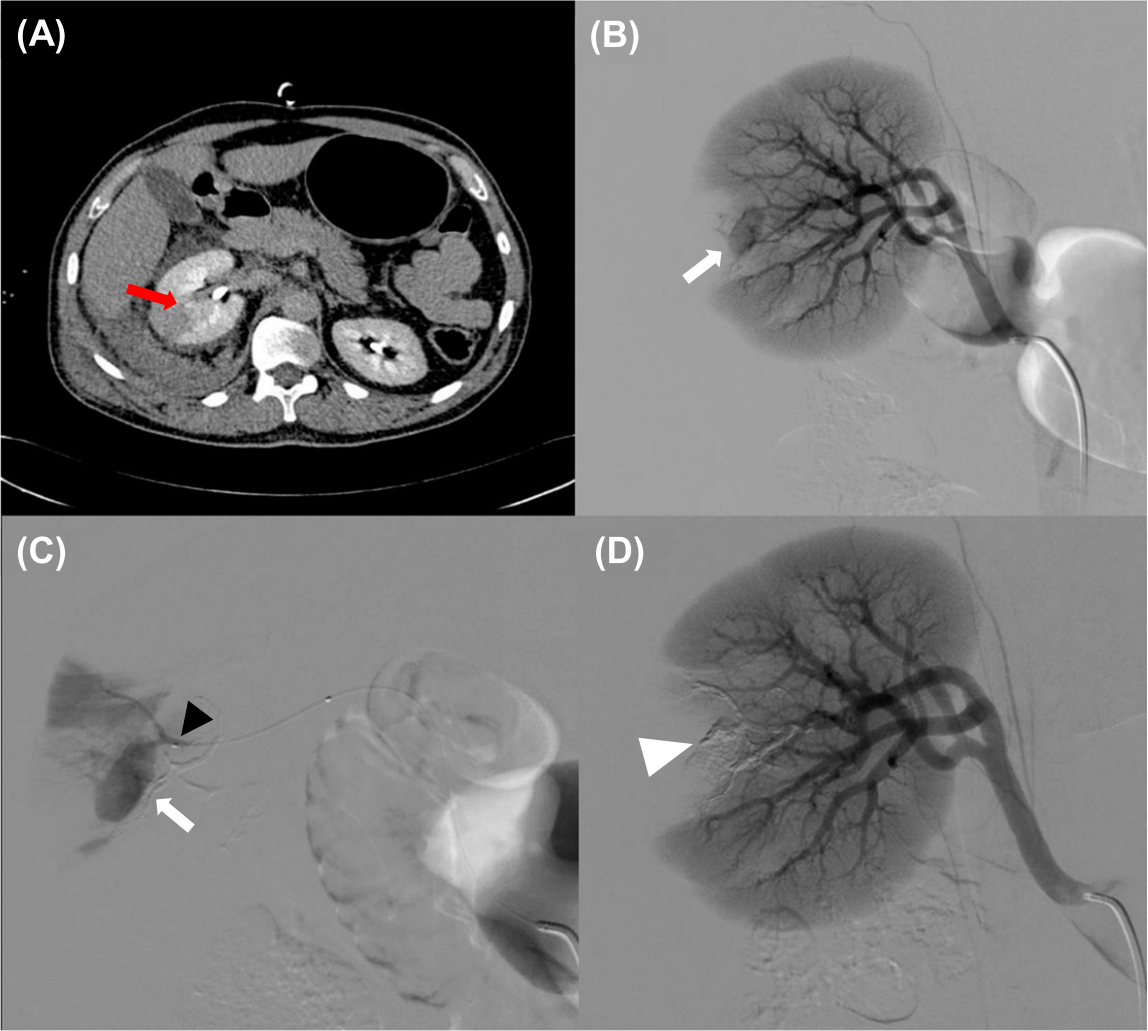
(**a**) Computed tomography **(**CT) image after endovenous contrast injection, depicting contrast deposition inside the right kidney. (**b**) Digital subtraction angiography (DSA) with injection from right renal artery showing pseudoaneurysm (white arrow), best seen in image (**c**) with a DSA performed by a microcatheter (black arrow) in the segmentar renal artery. (**d**) After embolization, glue cast (white arrow heads) in a subtracted image in the renal territory after embolization with complete superseletiva exclusion of the injured vessel from the circulation

The procedure was performed by a standard percutaneous transfemoral access with a 5-F sheath. Angiography of the aorta and of the suspected vessel were performed with 5-F catheters. All procedures were done under fluoroscopy and/or roadmap technique. A microcatheter (Excelsior SL-10,Stryker; Echelon 10, Medtronic; Progreat 2.7,Terumo) was advanced over a 0.014 in. wire (Transend-Stryker, or SilverSpeed-Medtronic) in order to approach the feeding artery of the hemorrhage as closed as possible (Fig. 1). Before embolization, the microcatheter was flushed with 5% dextrose solution followed by injection of NBCA (Glubran 2; GEM Srl, Viareggio, Italy or Histoacryl®; B. Braun, AG, Melsungen, Germany), in a solution with ethiodized oil (Lipiodol UF; Guerbet, Villepinte, France) ranging from 1:2 to 1:5 proportion, depending on the location of the injury. The microcateter was removed 1 to 3 min after embolization in order to avoid their imprisonment. A final control angiogram was performed in order to confirm occlusion of the bleeding arteries. Categorical variables were presented as percentages.

## Results

Forty-seven consecutive patients presenting with traumatic APB who underwent embolization were included in this study. The mean age of the patients in this study was 38.6 years (ranging from 3 to 81 years) with males forming 87.2% of them. The main cause of trauma was due to automobile accidents (70% of patients). Moreover, six patients had knife wounds, eight had falls from height (scaffolding, roofs, etc.) and one had undergone trampling by cattle. Among all abdominal bleeding, 18 were in liver, nine in spleen, three in kidney, one in adrenal and two in abdominal wall. Pelvic trauma accounted for 14 cases (29.8%). Two related to bladder rupture and the others to pelvic ring vascular lesions (rupture, stop and arterial parietal irregularity) (Table 1). Trauma was classified according to the criteria of the American Association of Trauma Surgery. In liver trauma, 23% of grade V, 54% of grade IV, 7.7% of grade III and 15.3% of grade II. The splenic traumas were divided into 12.5% grade V, 25% grade IV, 50% grade III and 12.5% grade II. Renal traumas were classified as grade IV and adrenal trauma as grade III.

**Table 1:** Clinical data of patients

Clinical data (N)	Total population (*N* = 47)
Age, years (median, range)	38.6 (3–81)
Male, (n, %)	41 (87.2)
Region of trauma (n, %)	
Abdominal	33 (70.2)
Pelvic	14 (29.8)
Potentially injured organs (n, %)	
Liver	18 (38)
Pelvis	14 (29.8)
Spleen	9 (18.9)
Kidney	3 (6.4)
Abdominal wall	2 (4.6)
Adrenal	1 (2.3)
Type of trauma (n, %)	
Automobile	32 (68)
Knife wounds	6 (12.7)
Fall from height	8 (17)
Others	1 (2.3)

**Fig. 2:**
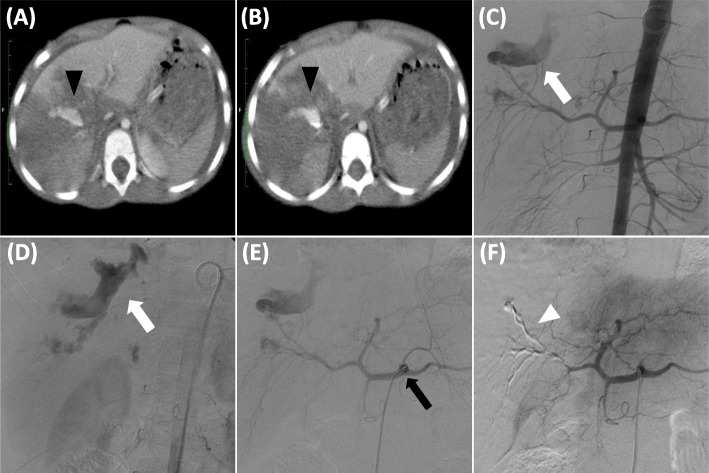
(**a, b**) CT image arterial phase after endovenous contrast injection, depicting contrast deposition inside the right liver lobe (black arrowhead). (**c, d**) DSA with Pigtail cateter injection in aorta showing contrast extravasation (white arrow), seen in image (**e**) with a DSA performed by a Cobra 2 catheter (black arrow) in the celiac trunk. (**f**) After embolization, glue cast (white arrowhead) in a subtracted image in the liver territory after embolization with complete superseletiva exclusion of the injured vessel from the circulation

Ninety-seven and a half percent of the cases underwent contrast-enhanced computer tomography (CT) and digital subtraction angiography (DSA). Only one patient did not undergo contrast-enhanced CT due to significant hemodynamic instability at the time of arrival at the institution, with no clinical conditions for open surgery, and a DSA was performed for diagnosis and therapy at the same time. The main CT finding was contrast extravasation (78.2% of the cases who underwent CT) (Fig. 2) and one case of arteriovenous fistula. In the other nine cases, no findings suggestive of active bleeding were observed on CT; however, alterations of the hematimetric levels in these patients were decisive factor in the indication of DSA. In DSA, contrast extravasation, contrast stagnation, arterial blush or anomalous blush, pseudoaneurysm, vascular irregularity/stop and arteriovenous fistula were observed with contrast extravasation being the most common of them (93,6%). In 78,2% of the cases, APB was observed on CT and confirmed by DAS in the same topography, while in the remaining 21.8% the findings related to active bleeding were observed solely by DSA (Table 2).

**Table 2:** Imaging data

	Total population (*N* = 47)
Admission (n, %)	
CT	46 (97,8)
DSA	1 (2,2)
DSA (n, %)	47 (100)
CT Finding	
Contrast Extravasation	36 (78,2)
Others	1 (6,4)
Without finding	9 (15,4)
DSA finding	
Contrast Extravasation	44 (93,6)
Others	3 (6,4)

*DSA* digital subtraction angiography, *CT* computed tomography

All procedures were performed with NBCA as the only embolic material. In all cases, only one microcatheter was used for occlusion of the bleeding site, and only one injection of NBCA solution was necessary. Lipiodol and NBCA solutions varied from 1:2 to 1:5 (NBCA:Lipiodol), depending on the location and type of the lesion. In 51% of all cases, we used a concentration of 1:3(25%), which, in this study, was considered as standard for a fast but still well controlled propagation of the embolic liquid. In topographies which access was difficult or too distal, we used a more diluted and fluid solution 1:4 (20%) or 1:5 (16.7%) to obtain a more distal propagation of the embolic fluid. In cases in which we consider as high-flow lesions, such as arteriovenous fistulas, we used 1:2 (33,3%) to avoid delayed polymerization and the propagation of embolic material to the venous side and then to non-target organs.

**Fig. 3:**
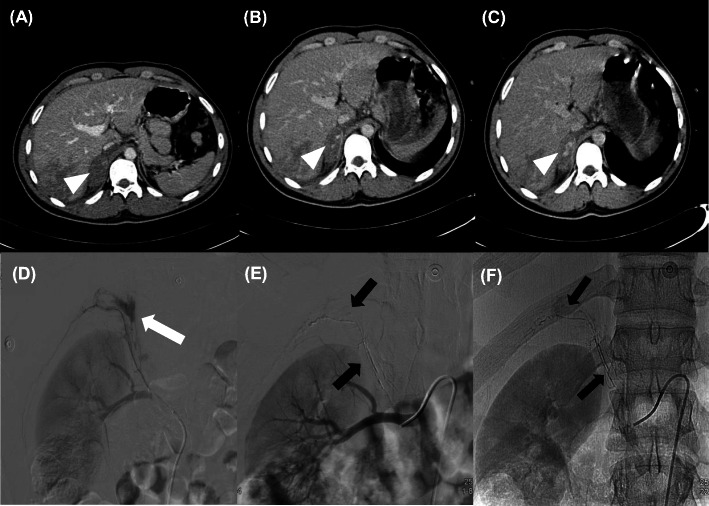
(**a, b, c**) CT image venous phase (**a**) and arterial phase (**b, c**) after endovenous contrast injection, depicting contrast deposition inside the right adrenal gland (white arrowhead). (**d**) DSA with injection right renal artery showing contrast extravasation (white arrow). (**e**) DSA with injection right adrenal artery showing glue cast (black arrow) in a subtracted image in the adrenal territory after embolization with complete superseletiva exclusion of the injured vessel from the circulation. (**f**) Subtraction image showing glue cast (black arrow)

We didn’t observe cases of non-target vascular occlusion via inadvertent embolization, which is considered one of the most serious clinically significant NBCA complication (Monsignore et al. 2012). In cases of abdominal wall bleeding embolization, were not observed any cutaneous necrosis. In all cases embolization was performed as distal as possible to the bleeding site, and in only one case a complete arterial organ occlusion was necessary, which was in an adrenal trauma (Fig. 3). We also didn’t observe vessel injuries such as spasm, dissection or perforation, microcatheter rupture and catheter entrapment.

Seven deaths (14.9%) occurred and were caused by other conditions such as clinical decompensation (mainly related to sepsis) or brain injuries, such as intracranial hemorrhage. No death was related to the endovascular procedure. Data analysis did not identify hemorrhagic or thromboembolic complications related to the embolization.

In this series the technical success rate was 100%, while no complications were observed related to the procedure (Table 3).

**Table 3:** Procedures and results

	Total population (*N* = 47)
Technical Success (n, %)	47 (100)
Clinical Success (n, %)	47 (100)
Embolic material	NBCA (100)
Complications (n, %)	0 (0)
Mortality (n, %)	7 (14,8)
Clinical decompensation	4 (57,1)
Brain injury	3 (42,9)

*NBCA* N-Butyl Cyanoacrylate

## Discussion

Trauma is a cause of lesions in intra-abdominal solid organs and pelvic structures, affecting most patients in young adulthood, being responsible for high morbidity and mortality rates (Azami-Aghdash et al. 2018; Gad et al. 2012). In agreement with other studies, these lesions are the main etiologies of acute active bleeding in abdominal and pelvic structures. Trauma has a great economic impact, being responsible for 12% of health care costs worldwide (American College of Surgeons - Trauma Commite 2012).

Abdominopelvic trauma can be divided into penetrating and contused, the latter being the most common (Karamercan et al. 2008). In our study, the most frequently injured solid abdominal organ includes liver, followed by spleen, and kidney, consistently with other articles (Matthes et al. 2003; Smith et al. 2005) .

Approaches for the treatment of traumatic lesions of abdominal and pelvic structures are based on radiological findings, according to the American Association for the surgery of trauma classification, and the patient’s clinical condition (The ATLS Subcommittee, American College of Surgeons’ Committee on Trauma, and the International ATLS working group, Chicago I 2013).

With the evolution of the technique, materials, experience of interventional radiologists, with better results and lower rates of complications, and its minimally invasive aspect, the treatment of APB by embolization has had a significant role in the emergency scenario.

Among the hepatic vascular lesions, about 85% are caused by trauma (Monsignore et al. 2012); more than 80% of the hepatic lesions related to trauma can be treated with non-surgical interventions, such as clinical/radiological control (Petrowsky et al. 2012; Leppaniemi et al. 2011; Cherian et al. 2016). Embolization represents a significant number in non-surgical therapy, especially in patients with grade IV and V liver trauma, with high rates of success, with 97% of technical success at the end of the procedure (Ierardi et al. 2016; van der Wilden et al. 2013; Stassen et al. 2012; Monnin et al. 2008).

The management of splenic lesions presents divergences in the different trauma centers around the world, which indicates a difficulty in the management of this type of lesion, being widely considered the non-surgical treatment. In most of the reviewed studies, the splenic lesion with embolization was treated using as embolic material metal spirals and PVA particles (Wallis et al. 2010; Ierardi et al. 2016; Raikhlin et al. 2008; Wahl et al. 2004; Hagiwara et al. 2004), in contrast to the present study, which had the NBCA glue as the embolic material of choice. The technical success rates of the main studies are high, with 89 to 95% (Ierardi et al. 2016; Wahl et al. 2004; Hagiwara et al. 2004; Wei et al. 2008; Sabe et al. 2009) of clinical success reported in the literature.

Embolization of active renal bleeding evidenced on CT is considered an adjunct treatment to the non-operative treatment of these lesions. Renal super selective embolization has great value in preserving the functionality of the remaining parenchyma, due to the poor collateral network observed in this organ (Ptohis et al. 2017). The success rates in the transarterial embolization literature are around 96%, while the complication rates are around 8% (Ierardi et al. 2016). The most reported material in the literature for embolization in the treatment of renal trauma are metal spirals (Matthes et al. 2003), while in this study the NBCA was the embolic material of choice.

Pelvic trauma account for a large proportion of cases of trauma. Most of the treatment protocols reported in the literature in relation to pelvic trauma are based on the “Control of orthopedic damage” (Zealley and Chakraverty 2010; Hoff et al. 2002), which recommends a rapid intervention focused on bleeding control and life-saving measures, due to the high degree of bleeding and mortality of this type of trauma. External fixation and direct surgical hemostasis are the measures initially performed in pelvic trauma, but embolization has had an increasing role in emergency hemostasis in this territory (Zealley and Chakraverty 2010). Embolization success rates are high, ranging from 85 to 100% in the literature, and similar rates are observed in this article. The main embolic agents described in the literature for this type of trauma are NBCA, PVA particles and micromoles, alone or in association, in contrast to this study in which all cases were treated with NBCA.

Adrenal lesion secondary to thoracoabdominal trauma is a very rare disease and is difficult to suspect clinically. The literature on the subject is rare, covered only by case reports (Fowler et al. 2013). Imaging exams performed at the admission of polytraumatized patients, such as CT, are helpful in the diagnosis. Treatment of this type of lesion varies with severity, and may be conservative, open surgery, or TE. This kind of treatment of adrenal trauma is rare, as well as its epidemiology; however, it can also be of high value, sparing the patient from open surgery when there is high physiological stress secondary to the trauma. The literature is not sufficiently substantiated to evaluate superiority in relation to the materials used (Fowler et al. 2013). We observed only one case of active bleeding of the adrenal gland after traumatic abdominal trauma and the NBCA was used as the embolizing agent of choice.

When all the abdominal and pelvic territories eligible for embolization of traumatic bleeding are considered, the literature outlines a wide range of embolic agents, including metal spirals, hemostatic gelatin (Gelfoam®), PVA particles and, more rarely, NBCA and non-adhesive liquid embolic agents like Onyx®. The selection of embolic material has as main criterion the experience and preference of the interventional radiologist (Papakostidis et al. 2012; Lopera 2010; Wallis et al. 2010; Monnin et al. 2008).

The NBCA is an embolic agent rarely seen in the literature for the treatment of bleeding of various etiologies. In the current study, embolization performed for the treatment of trauma-related active bleeding lesions were performed with NBCA glue exclusively.

As a liquid embolic material, the NBCA has the benefit of completely occluding a vessel and/or hemorrhagic injury, which is extremely selective, rapidly prepared and used, solving the active bleeding quickly, when used by professionals trained in its manipulation.

Even though there is the possibility of non-target embolization, the NBCA is still considered of highly safety and effectiveness in the treatment of traumatic hemorrhagic lesions. As this embolizing agent does not depend on the patient’s coagulation status, it can be safely be used in cases of severe coagulopathy, which is often observed in patients in the emergency room. Some interventional radiologists avoid the use of adhesive liquid embolic agents because they consider their manipulation and injection very difficult, probably due to lack of experience with the material. The main technical limitation of the use of NBCA is its manipulation. Its characteristics of polymerization, dilution with Lipiodol, injection speed, reflux control, anatomical familiarity are important aspects related to its use, and the physician should know them well to perform safe and successful procedures (Monsignore et al. 2012).

The main complications related to the use of the NBCA are the occlusion of non-target territory, either by mistaken interpretation of the anatomy, migration of embolic material, reflux of embolic material and opening of arterio-arterial anastomoses during injection, or by migration of embolic material into the venous system, which may cause pulmonary embolism or restriction of venous return (Niimi et al. 2003). The limitations of our study were the small number of patients, its retrospective nature and and the absence of a control group, although it was consistent with other reports in the literature.

## Conclusions

Endovascular embolization of traumatic abdominopelvic bleedings appears to be a highly safe and effective treatment, while avoiding emergent exploratory open surgeries.

## Data Availability

All data generated or analysed during this study are included in this published article and its supplementary information files.
